# Effectiveness of the BNT162b2 (Pfizer-BioNTech) and the ChAdOx1 nCoV-19 (Oxford-AstraZeneca) vaccines for reducing susceptibility to infection with the Delta variant (B.1.617.2) of SARS-CoV-2

**DOI:** 10.1186/s12879-022-07239-z

**Published:** 2022-03-20

**Authors:** Karan Pattni, Daniel Hungerford, Sarah Adams, Iain Buchan, Christopher P. Cheyne, Marta García-Fiñana, Ian Hall, David M. Hughes, Christopher E. Overton, Xingna Zhang, Kieran J. Sharkey

**Affiliations:** 1 Department of Mathematical Sciences, University of Liverpool, Liverpool, UK; 2 Centre for Global Vaccine Research, Institute of Infection, Veterinary and Ecological Sciences, University of Liverpool, Liverpool, UK; 3 Graphnet Health, Milton Keynes, UK; 4 Institute of Population Health, University of Liverpool, Liverpool, UK; 5 Department of Health Data Science, University of Liverpool, Liverpool, UK; 6 Department of Mathematics and School of Health Sciences, University of Manchester, Manchester, UK; 7 Joint Universities Pandemic and Epidemiological Research, Manchester, UK

**Keywords:** SARS-CoV-2, COVID-19, Vaccine, SIR, Modelling, Infectious disease, Effectiveness

## Abstract

**Background:**

From January to May 2021 the alpha variant (B.1.1.7) of SARS-CoV-2 was the most commonly detected variant in the UK. Following this, the Delta variant (B.1.617.2) then became the predominant variant. The UK COVID-19 vaccination programme started on 8th December 2020. Prior to the Delta variant, most vaccine effectiveness studies focused on the alpha variant. We therefore aimed to estimate the effectiveness of the BNT162b2 (Pfizer-BioNTech) and the ChAdOx1 nCoV-19 (Oxford-AstraZeneca) vaccines in preventing symptomatic and asymptomatic infection with respect to the Delta variant in a UK setting.

**Methods:**

We used anonymised public health record data linked to infection data (PCR) using the Combined Intelligence for Population Health Action resource. We then constructed an SIR epidemic model to explain SARS-CoV-2 infection data across the Cheshire and Merseyside region of the UK. Vaccines were assumed to be effective after 21 days for 1 dose and 14 days for 2 doses.

**Results:**

We determined that the effectiveness of the Oxford-AstraZeneca vaccine in reducing susceptibility to infection is 39% (95% credible interval [34, 43]) and 64% (95% credible interval [61, 67]) for a single dose and a double dose respectively. For the Pfizer-BioNTech vaccine, the effectiveness is 20% (95% credible interval [10, 28]) and 84% (95% credible interval [82, 86]) for a single-dose and a double dose respectively.

**Conclusion:**

Vaccine effectiveness for reducing susceptibility to SARS-CoV-2 infection shows noticeable improvement after receiving two doses of either vaccine. Findings also suggest that a full course of the Pfizer-BioNTech provides the optimal protection against infection with the Delta variant. This reinforces the need to complete the full course programme to maximise individual protection and reduce transmission.

**Supplementary Information:**

The online version contains supplementary material available at 10.1186/s12879-022-07239-z.

## Background

The UK COVID-19 vaccination programme started on the 8th December 2020 and, by 19 September 2021, the overall vaccine uptake for 1 dose was 89.3% and 83.9% for 2 doses in England for adults aged 18 and over [1]. Assessing the effectiveness of the vaccines is important for government policy, and particularly so as more transmissible variants of SARS-CoV-2 emerge [2]. Delta (B.1.617.2) was the dominant SARS-CoV-2 variant in the UK from June 2021 to mid-December 2021 [3, 4], and vaccine effectiveness studies at the time focused on this variant [5–7].

Direct vaccine effectiveness is often estimated using a test-negative case-control design, which compares the odds of vaccination in a group of symptomatic individuals that test positive for COVID-19 with the control group who are defined as individuals showing symptoms of COVID-19 but test negative. This methodology was employed in two recent COVID-19 vaccine effectiveness studies conducted in England and Scotland [5, 7]. A study by Pouwels et al., 2021 used a more traditional case-control design with survey data from randomly selected households across the UK [6]. Here, the control group consisted of randomly selected individuals who did not contract COVID-19. Test-negative designs are often logistically beneficial and cost-effective and can help to minimise selection bias because the cases and controls are assumed to have similar health-seeking behaviour. But, one of the issues raised with test-negative case-control designs is the lack of generalisability [8, 9]; that is, it only considers individuals who have sought to get tested and, therefore, findings may not be generalisable to those individuals who did not access testing services.

Vaccine effectiveness can also be estimated using a compartmental epidemic model that accounts for vaccination. Various methods of forecasting SARS-COV-2, including compartmental models, have thus far have been used to study hypothetical scenarios [10]. For example, Wong et al., 2021 used the SIR (susceptible, infected or removed/recovered) model to consider a single dose vaccination program and used it to project new SARS-CoV-2 cases based on different vaccine effectiveness levels [11]. Another example is the modified version of the SEIR (where E represents a compartment for exposed individuals) model with a single dose vaccine program, and waning natural and vaccine-induced immunity, which is used to study different vaccination policies [12].

Here we consider a modified SIR model with a multi-dose vaccine program to estimate vaccine effectiveness for the BNT162b2 (hereafter referred to as Pfizer-BioNTech) and ChAdOx1 nCoV-19 (hereafter referred to as Oxford-AstraZeneca) vaccines for reducing susceptibility to infection with respect to the Delta variant in England, UK. The method we consider here is not restricted to those individuals who have been tested but considers the entire resident population, therefore providing a more generalisable population estimate for vaccine effectiveness against infection. The method also explicitly accounts for the temporal variation in vaccination levels as well as levels of infection in the population, removing these as a potential source of bias. We exploit a specific time window where initially low levels of infection are being driven rapidly upwards by the emergence of the Delta variant, justifying the use of a simple SIR model.

## Methods

### Data

We used data from the Combined Intelligence for Population Health Action (CIPHA; www.cipha.nhs.uk) data resource. CIPHA covers the population health management of over 2.6M General Practice registered individuals of Cheshire and Merseyside, UK. It includes person-level linked anonymised records across the National Health Service (NHS), local government, social care, administrative and public health information systems. From CIPHA we have detailed case data for SARS-CoV-2 PCR positive individuals together with individual-level vaccination data. For demographics of data see Additional file 1: Table S1.1.

Demographic data and our denominator population was for Cheshire and Merseyside and was taken from the general practice registered population, sourced from the Spine Demographics service in North West England. SARS-CoV-2 PCR testing data came from the Public Health England (PHE) Second Generation Surveillance System (SGSS) feeds. For this work this consisted of all Pillar 1 (swab testing in PHE labs and NHS hospitals) and Pillar 2 (swab testing for the wider population, as set out in government guidance) tests taken by individuals whose home address was registered within Cheshire and Merseyside [13]. We considered only SARS-CoV-2 PCR positive cases in this study and vaccination status data came from the National Immunisation Management System (NIMS). All of these data feeds came via the CIPHA platform. To reduce testing exclusion, the UK government provided free PCR tests where individuals could order a PCR test kit to be sent to their home. Alternatively they could book an appointment at a walk-in or drive-through test site. However, under-reporting of SARS-CoV-2 cases is likely, and could be up to 50% [14]. We have therefore taken this into account in our modelling approach.

### SIR model with vaccination

**Fig. 1:**
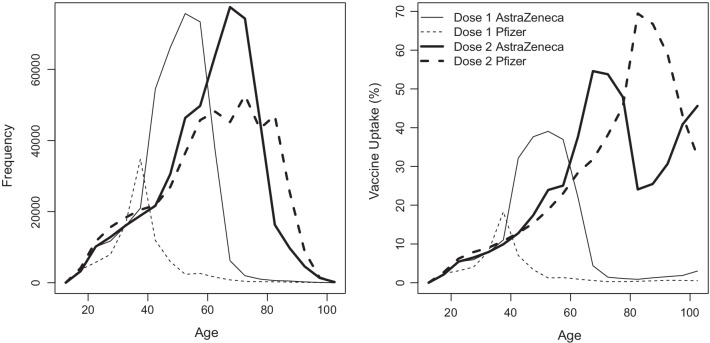
Histograms showing the age distribution for vaccination frequency and vaccination uptake for both Pfizer-BioNTech and Oxford-AstraZeneca vaccines on and before 24 May 2021 constructed using CIPHA data

**Fig. 2:**
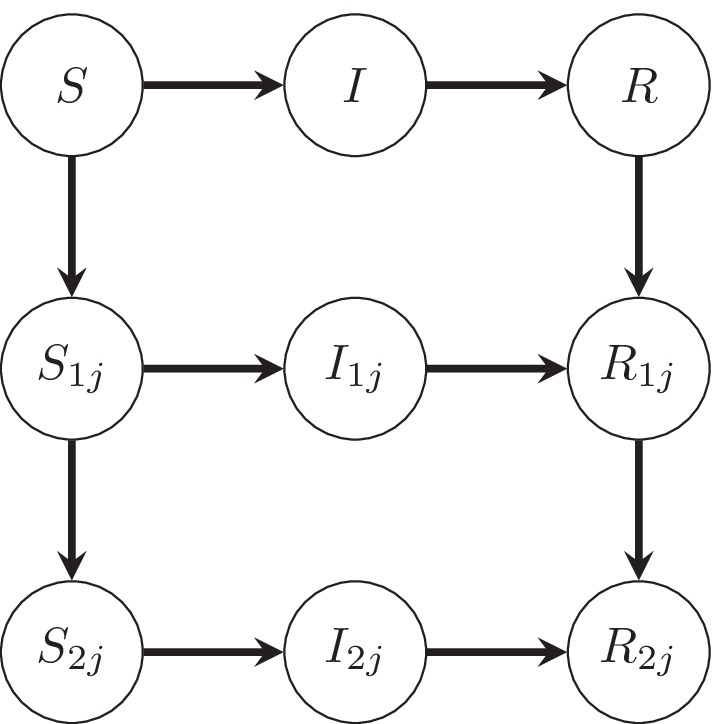
The transitions between the various classes of individuals, namely susceptible (*S* and $S_{ij}$), infected (*I* and $I_{ij}$) and removed (*R* and $R_{ij}$) when considering a two-dose vaccine program. *S*, *I*, *R* are unvaccinated and $S_{ij},I_{ij},R_{ij}$ are vaccinated where *i* is the number of doses received and *j* is the type of vaccine

We use an SIR model [15, 16] where individuals are also classified according to their vaccination status. We do not consider an age stratified model to keep parameters to a manageable level. There is some difference in age distribution between the Pfizer-BioNTech and Oxford-AstraZeneca vaccines (Fig. 1) but we consider this sufficiently small for this simplification to be used, particularly for two doses. Susceptible, infected and removed individuals are respectively denoted *S*, *I*, and *R* when unvaccinated, and $S_{ij}$, $I_{ij}$, and $R_{ij}$ when vaccinated, where *i* is the number of doses and *j* is the type of vaccine. Considering a two-dose vaccine program, i.e., $i\in \{1,2\}$, the flows between the various classes are shown in Fig. 2 and the system of differential equations is given by:

$$\begin{aligned} \frac{dS}{dt}&= -\beta S \frac{I+\sum _{i,j} \mu _iI_{ij}}{N} -\sum _jV_{1j}\frac{S}{S+R}\\ \frac{dS_{1j}}{dt}&= -(1-e_{1j})\beta S_{1j} \frac{I+\sum _{i,j} \mu _iI_{ij}}{N} \\&\quad +V_{1j}\frac{S}{S+R} -V_{2j}\frac{S_{1j}}{S_{1j}+R_{1j}}\\ \frac{dS_{2j}}{dt}&= -(1-e_{2j})\beta S_{2j} \frac{I+\sum _{i,j} \mu _iI_{ij}}{N} +V_{2j}\frac{S_{1j}}{S_{1j}+R_{1j}}\\ \frac{dI}{dt}&= \beta S \frac{I+\sum _{i,j} \mu _iI_{ij}}{N} -\gamma I\\ \frac{dI_{1j}}{dt}&= (1-e_{1j})\beta S_{1j} \frac{I+\sum _{i,j} \mu _iI_{ij}}{N} -\gamma I_{1j}\\ \frac{dI_{2j}}{dt}&= (1-e_{2j})\beta S_{2j} \frac{I+\sum _{i,j} \mu _iI_{ij}}{N} -\gamma I_{2j}\\ \frac{dR}{dt}&= \gamma I - \sum _jV_{1j}\frac{R}{S+R}\\ \frac{dR_{1j}}{dt}&= \gamma I_{1j} + V_{1j}\frac{R}{S+R} \\&\quad - V_{2j}\frac{R_{1j}}{S_{1j}+R_{1j}} \\ \frac{dR_{2j}}{dt}&= \gamma I_{2j} + V_{2j}\frac{R_{1j}}{S_{1j}+R_{1j}}. \end{aligned}$$

As shown in Fig. 2, the flow of individuals in this model is not only from susceptible to infected to removed, but also unvaccinated to one-dose to two-doses within the susceptible and removed classes. Birth and death processes are neglected in this model. The transmission rate, $\beta$, is the rate at which an unvaccinated infected individual (*I*) transmits the virus to an unvaccinated susceptible individual (*S*). For vaccinated infected individuals ($I_{ij}$), their infectiveness, i.e., how likely they are to infect a susceptible individual, is assumed to be reduced by a factor $\mu _i$ after *i* doses and we make no distinction between the vaccine types; this parameter is shown to be nonidentifiable [17], see “Results” section for more details. In vaccinated susceptible individuals ($S_{ij}$), the effectiveness of dose *i* of vaccine *j* in preventing infection is $e_{ij}$. The recovery rate, $\gamma$, of an infected individual is assumed to be the same regardless of their vaccination status. As can be seen from the form of the equations for infectious individuals, we expect this parameter to be highly correlated with $\beta$ and so not well-constrained by the data. We investigate sensitivity to this parameter over a wide range of plausible values and show that our conclusions on vaccine effectiveness are not sensitive to this. The quantity $V_{ij}$ is the rate of vaccination with dose *i* of vaccine *j* and is determined from the CIPHA data to give a daily vaccination rate. We assume that $V_{1j}$ is evenly distributed to individuals in classes *S* and *R*, $V_{2j}$ is evenly distributed to the individuals in classes $S_{1j}$ and $R_{1j}$, and that infected individuals do not receive the vaccine. The basic SIR model is recovered by initialising all vaccinated populations at zero and setting $V_{ij}=0$ for all *i*, *j*. A summary of the notation used is given in Table 1.

For simplicity, we do not include an exposed state in our model since this would increase the number of fit parameters, lose information and increase instability. Adding a short delay between infection and infectiousness is unlikely to impact the parameters of interest which only concern the rate of exponential growth of the infectious population and previous analysis has shown that the type of data we investigate is more reliably analysed using an SIR model rather than an SEIR model [18].

**Table 1:** Table of notation

Notation	Definition
$\beta$	Transmission rate
$\gamma$	Rate of recovery (days$^{-1}$)
$e_{ij}$	Percentage effectiveness against infection of *i* doses of vaccine *j*
$V_{ij}$	Daily number of individuals who have received dose *i* of vaccine *j*
*N*	Number of individuals in the population
$\mu _i$	Factor reducing infectiveness due to *i* doses

### Model fitting

**Fig. 3:**
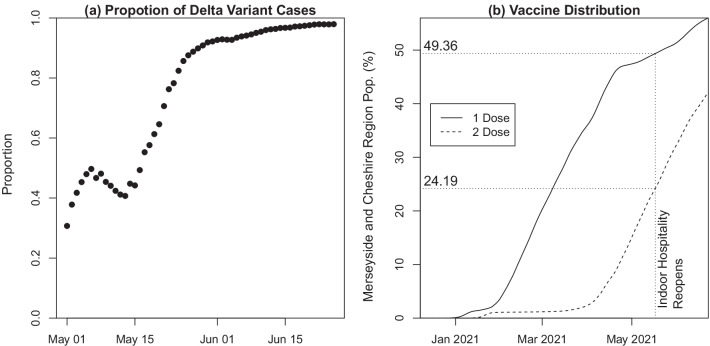
**a** Proportion of Delta variant cases from all SARS-CoV-2 PCR positive cases with known S-Gene target information for estimating variant status between 1st May 2021 and 25th June 2021. **b** Vaccine distribution in Cheshire and Merseyside region

On 17th May 2021, indoor hospitality was reopened in England. There was a spike in the number of covid-19 cases due to the dominance of the Delta variant in the Cheshire and Merseyside NHS region (see Fig. 3a) and across England [3] in combination with the lifting of restrictions. To construct Fig. 3a, a cycle threshold (Ct) cut-off of $\le 35$ [19] for S-gene, N-gene and ORF1ab (see [20] for details) is required to determine whether it is the Delta or another variant. Only infection data from processing labs that routinely looked at the 3 genes was used. At this point in time, 49% of the population had received 1 dose and 24% had received 2 doses in this region (see Fig. 3b). We use the rapid growth in infections during the period following this date to estimate the effectiveness of the Oxford-AstraZeneca and Pfizer-BioNTech vaccines in our model. During the period of our analysis, use of other vaccines was negligible (of the total vaccines administered, less than 2% of dose 1 and less than 1% of dose 2 were the Moderna vaccine in the CIPHA dataset and no other vaccine types were used) and there were no instances of individuals receiving two different vaccines.

**Fig. 4:**
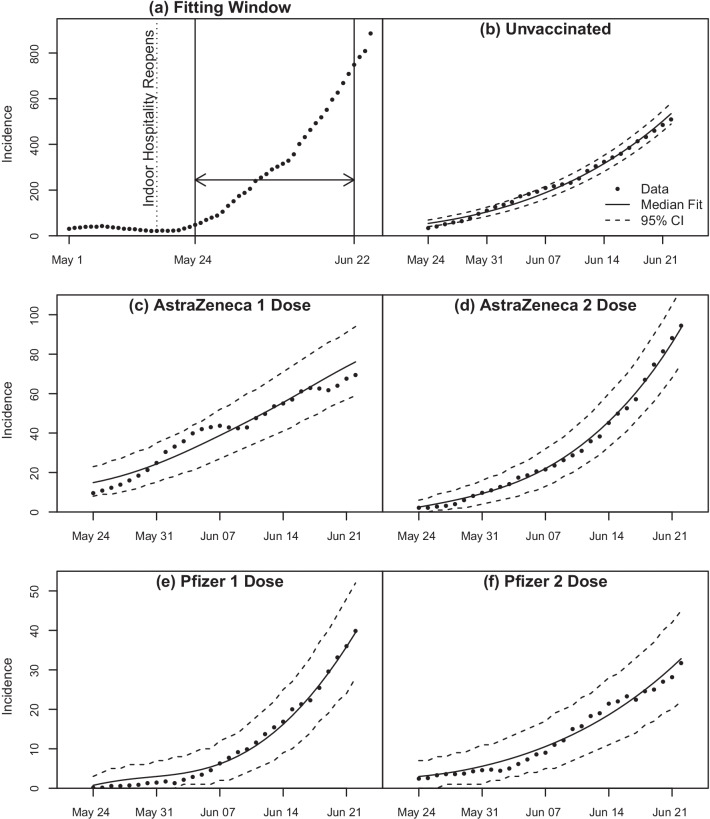
**a** Fitting window for 7-day rolling averaged data for all incidence. **b**–**f** SIR model with multi-dose vaccine and median parameter values from MCMC fitting analysis overlayed on 7-day rolling averaged incidence data for different vaccinations states

The fitting window used is shown in Fig. 4a. The fitting window starts on 24th May 2021, 7 days after indoor hospitality was reopened. This accounts for the delay in symptoms emerging, which is when people are likely to get tested for COVID-19 [21, 22] and also accounts for the 7 day symmetric rolling average, where the number of cases on a given day, 3 days before and 3 days after are averaged. We do this to smooth out the pronounced variations in reporting rates over the course of a week. We can have a symmetric rolling average since this is historic data but note that this differs from rolling averages computed for current data which necessarily involves the 6 preceding days [23].

Markov Chain Monte Carlo (MCMC) methods were used to fit to the incidence and vaccination time series data using the R-statistical package BayesianTools [24]. The values used to initialise the model fits are shown in Table 2. For parameters that are estimated by model fitting, a value was randomly chosen from their prior distribution to initiate the fits. An adaptive Metropolis-Hastings algorithm was used where the parameter covariance was updated every 500 iterations after a burn-in of 2000 iterations. The algorithm was run for total of $8\times 10^5$ iterations excluding burn-in. The final $3\times 10^5$ iterations were used to construct the posterior distributions of the parameters, which are plotted in the Additional file 1.

The likelihood function used for MCMC fitting is the Negative Binomial function as in [25]. The Negative Binomial probability mass function is

$$\begin{aligned} \text {NB}(k|n,p )={k+n-1\atopwithdelims ()n-1}p^n(1-p)^k. \end{aligned}$$

The following parameterisation is used:

$$\begin{aligned} p=\frac{1}{\theta }, \quad \quad \quad n(\mu ,\theta ) = \frac{\mu }{\theta -1}, \end{aligned}$$

where $\mu$ is the mean of the distribution and the variance is $\mu \theta$. Let ${\mathbf {I}}(t) = [I(t),I_{ij}(t)]$ for all *i*, *j* be the observed daily incidence on day *t* and $\hat{{\mathbf {I}}}(t,x)$ be the incidence generated by the model on day *t* for a given set of model parameters *x*. It is assumed that

$$\begin{aligned} {\mathbb {E}}[{\mathbf {I}}(t)]=\hat{{\mathbf {I}}}(t,\theta ). \end{aligned}$$

The log likelihood function is then given by

$$\begin{aligned} L({\mathbf {I}}|x,\theta ) = \sum _t \ln \left[ \text {NB}\left( {\mathbf {I}}(t)|\hat{{\mathbf {I}}}(t,x)/(\theta -1),1/\theta \right) \right] . \end{aligned}$$

The SIR model with multi-dose vaccines is fitted to the data to estimate the posterior distributions of the transmission rate ($\beta$), the effectiveness of the vaccines ($e_{ij}$), the initial values of the infected classes (*I* and $I_{ij}$), and infectiousness ($\mu _i$). We have $i\in \{1,2\}$ and $j\in \{{A,P}\}$ for Oxford-AstraZeneca and Pfizer-BioNTech respectively, and therefore posterior distributions for 12 parameters are estimated by the model fit. For all model fits we use uniformly distributed priors for all 12 parameters estimated as shown in Table 2a. For the impact of vaccines on infectiousness ($\mu _i$), which implements the reduction in the ability of an individual to transmit the virus, we have no data for the Delta variant of COVID-19. For the Alpha variant, this has been estimated to be 0.45–0.50 for one dose of Pfizer-BioNTech and 0.35–0.50 for one dose of Oxford-AstraZeneca, with no data available for the second dose [26]. We therefore make no *a priori *assumption about infectiousness and consider its full range for the prior distribution; i.e., $\text {Unif}[0,1]$, allowing for analysis of sensitivity to these parameters. For vaccine effectiveness ($e_{ij}$), we also consider the full viable range for the prior distribution; i.e., $\text {Unif}[0,1]$. The prior distribution for the transmission rate ($\beta$) is $\text {Unif}[0,10]$. This takes into account that $\beta$ cannot be negative and we expect it to be lower than 10 as this is an extreme scenario where all individuals could get infected due to a high transmission rate. The prior distribution for initial *I* and initial $I_{ij}$ is $\text {Unif}[0,100]$. This takes into account that they cannot be negative and we expect the initial number of infected individuals in each group to be within 100 as the start of our fitting window is shortly after indoor hospitality was reopened (see Fig. 4a).

**Table 2:** Prior distributions and initial values (obtained from CIPHA data) used for model fits

(a) Prior distributions used for initialising model fits									
Name	$\bf {\gamma}$	Prior distributions			Initial values				
		$\bf {\beta}$	$\bf {I,I_{ij}}$	$\bf {e_{ij},\mu _{i}}$	$\bf {S,S_{ij},R,R_{ij}}$				
Core Model	1/7	$\text {Unif}[0,10]$	$\text {Unif}[0,100]$	$\text {Unif}[0,1]$	Table 2b				
Sensitivity to $\gamma$	1/3	$\text {Unif}[0,10]$	$\text {Unif}[0,100]$	$\text {Unif}[0,1]$	Table 2b				
Sensitivity to $\gamma$	1/11	$\text {Unif}[0,10]$	$\text {Unif}[0,100]$	$\text {Unif}[0,1]$	Table 2b				
Double Removed	1/7	$\text {Unif}[0,10]$	$\text {Unif}[0,100]$	$\text {Unif}[0,1]$	Table 2c				

(b) Initial values when assuming all infected from start of pandemic moved to removed class									
*S*	$\bf {S_{1A}}$	$\bf {S_{2A}}$	$\bf {S_{1P}}$	$\bf {S_{2P}}$	*R*	$\bf {R_{1A}}$	$\bf {R_{2A}}$	$\bf {R_{1P}}$	$\bf {R_{2P}}$
1266979	478347	312594	74665	384481	70887	42715	20895	6738	26715

(c) Initial values when assuming infected individuals are underestimated by 50%									
*S*	$\bf {S_{1A}}$	$\bf {S_{2A}}$	$\bf {S_{1P}}$	$\bf {S_{2P}}$	*R*	$\bf {R_{1A}}$	$\bf {R_{2A}}$	$\bf {R_{1P}}$	$\bf {R_{2P}}$
1196092	435632	291699	67927	357766	141774	85430	41790	13476	53430

Notation is as follows $\beta$: Transmission rate, $\gamma$: Recovery rate, $e_{ij}$: Effectiveness of *i* doses of vaccine *j* against infection, *S*: Unvaccinated susceptible individuals, $S_{ij}$: Susceptible individuals who have received *i* doses of vaccine *j*, *I*: Unvaccinated infected individuals, $I_{ij}$: Infected individuals who have received *i* doses of vaccine *j*, *R*: Unvaccinated removed individuals, $R_{ij}$: Removed individuals who have received *i* doses of vaccine *j*, and $\mu _i$: Infectiveness of an infected individual with *i* doses of either vaccine. For number of doses we have $i\in \{1,2\}$ and for vaccines $j\in \{A,P\}$ for Oxford-AstraZeneca and Pfizer-BioNTech respectively

The infectious period in days is given by $1/\gamma$, with estimates ranging between 3 and 11 days according to [27]. We therefore consider three model fits with regards to $\gamma$ (see Table 2a). For our ‘Core Model’ fit we use a median infectious period of 7 days, i.e., $\gamma =1/7$. To account for sensitivity to the infectious period, we also fit the model for $\gamma =1/3$ and $\gamma =1/11$; these fits are called ‘Sensitivity to $\gamma$’. For these three fits, the fixed parameters ($V_{ij}$, *N*) and the initial values of $S,S_{ij},R,R_{ij}$, are obtained from CIPHA data. For $V_{ij}$ we take into account a lag for the vaccine to come into effect. An individual is assumed to move into the relevant vaccinated category after a delay of 21 days post-vaccination for dose 1 and 14 days post-vaccination for dose 2 of either vaccine [28]. From the CIPHA database, the population size covering Cheshire and Merseyside is 2,730,111 (Additional file 1: Table S1.1). On 24th May 2021, we obtain $N=2,691,418$ after removing deceased individuals from any cause. We assume this number is fixed for the duration of the fit window. The initial values $S_{ij}$ and $R_{ij}$ are shown in Table 2b. The number removed are taken from all individuals who have ever been recorded as infected since the beginning of the pandemic, except for those that died. We are therefore assuming infected individuals retain immunity for the remaining duration of the pandemic, and we are assuming that there is no under-reporting of cases (which is certainly not true, especially during the first wave of the pandemic) [14, 29]. The first of these assumptions leads to an overestimate of the removed category on 24th May 2021, and the second (likely more questionable) assumption leads to an underestimate of the removed category. We demonstrate insensitivity of our conclusions to these assumptions by re-running our analysis assuming that only half of all the infected were actually detected overall, leading to a doubling of the initial removed category on 24th May 2021. This fit is called ‘Double Removed’ (see Table 2c).

## Results

Our model used a total population of 2,691,418. From the Core Model, the effectiveness against infection obtained for one dose is 38.5% (95% credible interval [34.3, 42.6]) for Oxford-AstraZeneca and 19.5% (95% credible interval [10.4, 28.1]) for Pfizer-BioNTech. For two doses, we obtained an effectiveness against infection of 64.0% (95% credible interval [61.4, 66.5]) for Oxford-AstraZeneca and 83.9% (95% credible interval [82.1, 85.6]) for Pfizer-BioNTech. The median value and and 95% credible interval for all fitted parameters are shown in Additional file 1: Table S2.1 for all model fits. In Table 3, these values are shown for the Core Model except for the infectiveness of an infected individual with *i* doses of either vaccine ($\mu _i$) as they are nonidentifiable [17]. In particular, the trace plots for all parameters except $\mu _i$ converge (the MCMC trace plots and posterior distributions for all model fits are shown in the Additional file 1: Figs. S2.1–S2.4). We therefore constructed the log likelihood profiles for $\mu _i$ and they were flat (see Additional file 1: Fig. S3.1). This means they do not provide any information, confirming nonidentifiability.

Figure 4b–f shows the Core Model fit to data. The incidence curves and 95% confidence interval (CI) bands are plotted using the Core Model median parameter values (Table 3) together with the incidence curves given by the data. The 95% CI is generated by using the fact that the likelihood function used for MCMC fitting is the Negative Binomial function.

For all model fits (see Table 2a), the parameters of direct interest (the vaccine effectiveness parameters) are reproduced in Table 4 where other results from existing studies are reproduced for comparison. For our results we have stated the median value together with the 95% credible interval, where as for the results in [5–7] the mean value together with the 95% confidence interval is given.

**Table 3:** Estimates obtained from Core Model ($\gamma =1/7$)

Parameter	Median (50%)	Lower (2.5%)	Upper (97.5%)
$\beta$	0.4060	0.3893	0.4425
$e_{1A}$	0.3851	0.3425	0.4260
$e_{2A}$	0.6402	0.6140	0.6647
$e_{1P}$	0.1954	0.1041	0.2809
$e_{2P}$	0.8392	0.8212	0.8559
Initial *I*	54.0151	48.4328	59.5435
Initial $I_{1A}$	14.8932	10.3012	19.8382
Initial $I_{2A}$	2.4775	0.9300	4.7359
Initial $I_{1P}$	0.7469	0.1234	2.0681
Initial $I_{2P}$	2.9717	1.3856	5.1267

Notation is as follows $\beta$: Transmission rate, $e_{ij}$: Effectiveness of *i* doses of vaccine *j* against infection, Initial *I*: Initial number of unvaccinated infected individuals, Initial $I_{ij}$: Initial number of infected individuals who have received *i* doses of vaccine *j*. For number of doses we have $i\in \{1,2\}$ and for vaccines $j\in \{A,P\}$ for Oxford-AstraZeneca and Pfizer-BioNTech respectively

**Table 4:** Vaccine effectiveness against Delta variant and vaccine efficacy (clinical trials carried out prior to Delta strain being detected)

References	Infection	Delay		Dose 1 Effectiveness		Dose 2 Effectiveness	
		Dose 1	Dose 2	Oxford-AstraZeneca	Pfizer-BioNTech	Oxford-AstraZeneca	Pfizer-BioNTech
$$\begin{array}{c}\displaystyle \text {Core Model}\\ \displaystyle (\gamma =1/7)\end{array}$$	Sym. & Asym.	21	14	$$\begin{array}{c}\displaystyle 38.5\%\\ \displaystyle [34.3,42.6]\end{array}$$	$$\begin{array}{c}\displaystyle 19.5\%\\ \displaystyle [10.4,28.1]\end{array}$$	$$\begin{array}{c}\displaystyle 64.0\%\\ \displaystyle [61.4,66.5]\end{array}$$	$$\begin{array}{c}\displaystyle 83.9\%\\ \displaystyle [82.1,85.6]\end{array}$$
Bernal et al. [5]	Sym.	21	14	$$\begin{array}{c}\displaystyle 30.0\%\\ \displaystyle (24.3,35.3)\end{array}$$	$$\begin{array}{c}\displaystyle 35.6\%\\ \displaystyle (22.7,46.4)\end{array}$$	$$\begin{array}{c}\displaystyle 67.0\%\\ \displaystyle (61.3,71.8)\end{array}$$	$$\begin{array}{c}\displaystyle 88.0\%\\ \displaystyle (85.3,90.1)\end{array}$$
Pouwels et al. [6]	Sym. & Asym.	21	14	$$\begin{array}{c}\displaystyle 46\%\\ \displaystyle (35,55)\end{array}$$	$$\begin{array}{c}\displaystyle 57\%\\ \displaystyle (50,63)\end{array}$$	$$\begin{array}{c}\displaystyle 67\%\\ \displaystyle (62,71)\end{array}$$	$$\begin{array}{c}\displaystyle 80\%\\ \displaystyle (77,83)\end{array}$$
Sheikh et al. [7]	Sym. & Asym.	28	14	$$\begin{array}{c}\displaystyle 33\%\\ \displaystyle (23,41)\end{array}$$	$$\begin{array}{c}\displaystyle 33\%\\ \displaystyle (15,47)\end{array}$$	$$\begin{array}{c}\displaystyle 61\%\\ \displaystyle (51,70)\end{array}$$	$$\begin{array}{c}\displaystyle 83\%\\ \displaystyle (78,87)\end{array}$$
$$\begin{array}{c}\displaystyle \text {Sensitivity to } \gamma \\ \displaystyle (\gamma =1/11)\end{array}$$	Sym. & Asym.	21	14	$$\begin{array}{c}\displaystyle 40.8\%\\ \displaystyle [36.2,45.1]\end{array}$$	$$\begin{array}{c}\displaystyle 14.4\%\\ \displaystyle [4.52,23.5]\end{array}$$	$$\begin{array}{c}\displaystyle 62.5\%\\ \displaystyle [59.6,65.1]\end{array}$$	$$\begin{array}{c}\displaystyle 83.7\%\\ \displaystyle [81.8,85.5]\end{array}$$
$$\begin{array}{c}\displaystyle \text {Sensitivity to } \gamma \\ \displaystyle (\gamma =1/3)\end{array}$$	Sym. & Asym.	21	14	$$\begin{array}{c}\displaystyle 35.3\%\\ \displaystyle [31.2,39.2]\end{array}$$	$$\begin{array}{c}\displaystyle 26.3\%\\ \displaystyle [18.1,34]\end{array}$$	$$\begin{array}{c}\displaystyle 66.2\%\\ \displaystyle [63.8,68.4]\end{array}$$	$$\begin{array}{c}\displaystyle 84.2\%\\ \displaystyle [82.5,85.8]\end{array}$$
$$\begin{array}{c}\displaystyle \text {Double Removed}\\ \displaystyle (\gamma =1/7)\end{array}$$	Sym. & Asym.	21	14	$$\begin{array}{c}\displaystyle 36.4\%\\ \displaystyle [31.9,40.6]\end{array}$$	$$\begin{array}{c}\displaystyle 18.3\%\\ \displaystyle [9.14,26.9]\end{array}$$	$$\begin{array}{c}\displaystyle 63.3\%\\ \displaystyle [60.7,65.8]\end{array}$$	$$\begin{array}{c}\displaystyle 83.7\%\\ \displaystyle [81.8,85.3]\end{array}$$
				Dose 1 Efficacy		Dose 2 Efficacy	
Polack et al. [30]	Sym.	12	7	–	$$\begin{array}{c}\displaystyle 52\%\\ \displaystyle [29.5,68.4]\end{array}$$	–	$$\begin{array}{c}\displaystyle 95\%\\ \displaystyle [90.3,97.6]\end{array}$$
Voysey et al. [31]	Sym.	21	14	$$\begin{array}{c}\displaystyle 64.1\%\\ \displaystyle [50.5,73.9]\end{array}$$	–	$$\begin{array}{c}\displaystyle 70.4\%\\ \displaystyle [54.8,80.6] \\ \text {95.8\% credible interval}\end{array}$$	–

Delay is the number of days after which the effectiveness or efficacy is measured, and $\gamma$ is the recovery rate. 95% credible interval shown in square brackets $[\ ]$ and 95% confidence interval shown in parentheses $(\ )$, unless stated otherwise. The infection column specifies whether infection is symptomatic and asymptomatic ( Sym. & Asym.) or symptomatic (Sym.)

## Discussion

We assessed the effectiveness of the Oxford-AstraZeneca and Pfizer-BioNTech vaccines in reducing the susceptibility of individuals to symptomatic and asymptomatic infection with respect to the SARS-CoV-2 Delta variant of COVID-19 using data from the Cheshire and Merseyside NHS region of the UK. We confirmed that both vaccines provide good protection after two doses but substantially less protection after one dose. The one dose effectiveness against infection was greater for Oxford-AstraZeneca (39%) compared to Pfizer-BioNTech (20%), however the Pfizer-BioNTech vaccine provides greater protection against infection with the Delta variant after two doses (84% compared to Oxford-AstraZeneca vaccine 64%). Our estimates of vaccine effectiveness against infection for one dose of Oxford-AstraZeneca and two-doses of either vaccine are consistent with those reported by [5–7] (Table 4). Even after changing assumptions, which include the infectious period and the number of removed individuals, the results are still consistent. Furthermore, all studies in Table 4 report a lower effectiveness when compared to the efficacy reported in clinical trials [30, 31] prior to the Delta variant being detected. This suggests that the Delta variant is better at evading vaccine induced immunity.

However, for one dose of the Pfizer-BioNTech vaccine, our estimate is slightly lower than that reported in these studies. One of the reasons for this is that in our study this group of individuals is much smaller than those groups who have received one dose of Oxford-AstraZeneca or two doses. The estimate is still comparable to those reported by Bernal and et al., 2021 and Sheikh et al., 2021 [5, 7]. Similar to our work, these studies included cases who had actively sought COVID-19 testing. In contrast, the study conducted by Pouwels et al., 2021 used a community household testing survey to identify cases and controls and notably reported higher 1st dose effect estimates for the Pfizer-BioNTech vaccine [6]. Note that this is also the case when compared to the clinical trial results reported in Polack et al., 2020 [30], where the efficacy reported is lower for the 1st dose of the Pfizer-BioNTech vaccine.

Here the effectiveness of the Oxford-AstraZeneca and Pfizer-BioNTech vaccines was estimated by fitting an SIR model where each class of individual was stratified by the number of doses and type of vaccine received. We identified a unique time period in May and June 2021 where the epidemic was undergoing exponential growth from a very low level due to the emergence of the Delta variant and lifting of restrictions, enabling the use of a simple SIR model. During this same period, substantial numbers of vaccines were being administered and this enabled us to extract strong signals for the effectiveness of single and double doses of the Pfizer-BioNTech and Oxford-AstraZeneca vaccines.

The temporal dynamics in each vaccination category are fully accounted for, removing biases caused by the interaction of vaccination rates/ types and the level of infection in the community. For studies employing case-control methodology, these biases are harder to account for, for example, there is an assumption that the vaccine under study has no effect on disease incidence in the control population (i.e., the herd effects) [7]. In addition, our study is not restricted to individuals who have sought to get tested and so minimises the issue of population level generalisability [8, 9].

A major assumption in our model is that the number of removed individuals at the start of the fitting window is given by the actual number of recorded infections throughout the whole pandemic. This could be problematic for two reasons. Firstly, there may be waning immunity of previously infected individuals. Secondly, there may be under-reporting of infections, particularly in the early stages of the pandemic. This could be due to asymptomatic infection, choosing not to get tested, due to lack of availability of testing or the change over time in the uptake of lateral flow testing as the preferred asymptomatic testing route. The two effects act in opposite directions and the second is likely to be the most dominant on the present timescale. This means that the number of susceptible individuals is likely to be overestimated. To account for this, we considered the case where the number of removed individuals in each class are doubled, representing an under-reporting of 50% [14]. This reduces the number in each respective susceptible class for the initial conditions of our model. We make no similar assumption for the dynamics of infection during our fitting window, since detection rates were likely to be high and the fit parameters of interest (exponential rates) are insensitive to reporting rates provided that these rates are constant during the fit window. The vaccine effectiveness estimates are found to be quite insensitive to even this significant modification.

### Limitations

It is important to note that our model does not stratify the population by age and therefore it does not take into account the effects of age on vaccine effectiveness. In the UK, vaccines were initially prioritised for the most vulnerable people and then distributed in decreasing order of age [32]. In the fitting window we have used to estimate vaccine effectiveness, unvaccinated individuals or individuals with one dose are much younger. In particular, there is a greater distribution of one dose amongst those ≤ 50 years, and a greater distribution of 2 doses amongst those ≥ 70 years. This means that the single dose vaccine effectiveness is likely to be biased towards the younger population, whereas those with two doses towards the older population. There may be an effect due to variation in immunity across age groups, where younger individuals are likely to have a better immune response to vaccines. The effectiveness of a given vaccine would therefore also depend upon how it is distributed across different age groups. However, Cheshire and Merseyside has had slower population level COVID-19 vaccine uptake compared to other areas of the UK [1], which has benefits for estimating vaccine effectiveness in post-licensure studies as this has resulted in a more heterogeneous age distribution.

## Conclusion

Vaccine effectiveness for reducing susceptibility to SARS-CoV-2 Delta variant infection shows noticeable improvement after receiving two doses of either vaccine. Our findings also suggest that a full course of the Pfizer-BioNTech vaccine provides the optimal protection against infection with the Delta variant. These findings advocate for completion of the full course to maximise individual protection and reduce transmission.

## Supplementary Information

**Additional file 1.** Population demographics, MCMC output (parameter estimates, trace plots and posterior distributions) for all models and log likelihood profile for infectiveness.

## Data Availability

Pseudonymised data are accessible via Combined Intelligence for Population Health Action (CIPHA). Requests can be made to the Data Asset and Access Group for extracts of the larger-scale data which cannot be released openly due to information governance requirements. All R code is accessible from the corresponding author.

## Abbreviations

AIC: Akaike information criterion
CI: Confidence interval
CIPHA: Combined intelligence for population health action
MCMC: Markov chain Monte Carlo
NHS: National Health Service
NIMS: National immunisation management system
PCR: Polymerase chain reaction
RNA: Ribose nucleic acid
SARS-CoV-2: Severe acute respiratory syndrome coronavirus 2
SIR: Susceptible, infectious or recovered/removed
SEIR: Susceptible, exposed, infectious or recovered/removed
UK: United Kingdom

## References

[1] COVID-19 Vaccination Statistics. Technical Report Week ending Sunday 3rd October 2021, NHS, 2021.

[2] Campbell F, Archer B, Laurenson-Schafer H, Jinnai Y, Konings F (2021). Increased transmissibility and global spread of SARS-CoV-2 variants of concern as at June 2021. Eurosurveillance.

[3] Torjesen I (2021). Covid-19: delta variant is now UK’s most dominant strain and spreading through schools. BMJ.

[4] SARS-CoV-2 variants of concern and variants under investigation in England. Technical Briefing 35, UK Health Security Agency, January 2022.

[5] Lopez Bernal J, Andrews N, Gower C, Gallagher E, Simmons R (2021). Effectiveness of Covid-19 Vaccines against the B.1.617.2 (Delta). N Engl J Med Variant.

[6] Pouwels KB, Pritchard E, Matthews PC, Stoesser N, Eyre DW (2021). Effect of Delta variant on viral burden and vaccine effectiveness against new SARS-CoV-2 infections in the UK. Nat Med.

[7] Sheikh A, McMenamin J, Taylor B, Robertson C (2021). SARS-CoV-2 Delta VOC in Scotland: demographics, risk of hospital admission, and vaccine effectiveness. The Lancet.

[8] Sullivan SG, Tchetgen Tchetgen EJ, Cowling BJ (2016). Theoretical basis of the test-negative study design for assessment of influenza vaccine effectiveness. Am J Epidemiol.

[9] Westreich D, Hudgens MG (2016). Invited commentary: beware the test-negative design. Am J Epidemiol.

[10] Bertozzi AL, Franco E, Mohler G, Short MB, Sledge D (2020). The challenges of modeling and forecasting the spread of COVID-19. Proc Natl Acad Sci.

[11] Wong W, Juwono FH, Chua TH. Sir simulation of Covid-19 pandemic in Malaysia: Will the vaccination program be effective? *arXiv preprint* arXiv:2101.07494, 2021.

[12] Acuña-Zegarra MA, Díaz-Infante S, Baca-Carrasco D, Olmos-Liceaga D (2021). COVID-19 optimal vaccination policies: a modeling study on efficacy, natural and vaccine-induced immunity responses. Math Biosci.

[13] COVID-19 testing data: methodology note. www.gov.uk/government/publications/coronavirus-covid-19-testing-data-methodology/covid-19-testing-data-methodology-note.

[14] Brereton C, Pedercini M (2021). COVID-19 case rates in the UK: modelling uncertainties as lockdown lifts. Systems.

[15] Kermack WO, McKendrick AG (1927). A contribution to the mathematical theory of epidemics. Proc Royal Soc A Math, Phys Eng Sci.

[16] Keeling MJ, Rohani P (2011). Modeling Infectious Diseases in Humans and Animals.

[17] Rannala B (2002). Identifiability of parameters in MCMC Bayesian inference of phylogeny. Syst Biol.

[18] Roda WC, Varughese MB, Han D, Li MY (2020). Why is it difficult to accurately predict the COVID-19 epidemic?. Infect Dis Model.

[19] Mishra B, Ranjan J, Purushotham P, Saha S, Payal P (2021). High proportion of low cycle threshold value as an early indicator of COVID-19 surge. J Med Virol.

[20] Udugama B, Kadhiresan P, Kozlowski HN, Malekjahani A, Osborne M (2020). Diagnosing COVID-19: the disease and tools for detection. ACS Nano.

[21] Tindale LC, Stockdale JE, Coombe M, Garlock ES, Lau WYV (2020). Evidence for transmission of COVID-19 prior to symptom onset. Elife.

[22] Crozier A, Rajan S, Buchan I, McKee M (2021). Put to the test: use of rapid testing technologies for Covid-19. BMJ.

[23] Roser M, Ritchie H, Ortiz-Ospina E, Hasell J. Coronavirus pandemic (COVID-19). Our world in data, 2020.

[24] Hartig F, Minunno F, Paul S. BayesianTools: general-purpose MCMC and SMC samplers and tools for Bayesian statistics, 2019.

[25] Pellis L, Scarabel F, Stage HB, Overton CE, Chappell LHK (2021). Challenges in control of COVID-19: short doubling time and long delay to effect of interventions. Philos Trans Royal Soc B Biol Sci.

[26] COVID-19 Vaccine Surveillance Report—Week 38. Technical report, Public Health England (PHE), 2021.

[27] Byrne AW, McEvoy D, Collins AB, Hunt K, Casey M (2020). Inferred duration of infectious period of SARS-CoV-2: rapid scoping review and analysis of available evidence for asymptomatic and symptomatic COVID-19 cases. BMJ Open.

[28] Pritchard E, Matthews PC, Stoesser N, Eyre DW, Gethings O (2021). Impact of vaccination on new SARS-CoV-2 infections in the United Kingdom. Nat Med.

[29] Lau H, Khosrawipour T, Kocbach P, Ichii H, Bania J (2021). Evaluating the massive underreporting and undertesting of COVID-19 cases in multiple global epicenters. Pulmonology.

[30] Polack FP, Thomas SJ, Kitchin N, Absalon J, Gurtman A (2020). Safety and Efficacy of the BNT162b2 mRNA Covid-19 Vaccine. N Engl J Med.

[31] Voysey M, Clemens SAC, Madhi SA, Weckx LY, Folegatti PM (2021). Safety and efficacy of the ChAdOx1 nCoV-19 vaccine (AZD1222) against SARS-CoV-2: an interim analysis of four randomised controlled trials in Brazil, South Africa, and the UK. Lancet (London, England).

[32] Joint Committee on Vaccination and Immunisation. Advice on priority groups for COVID-19 vaccination, 30 December 2020. Joint Committee on Vaccination and Immunisation: Technical report; 2021.

