# Supplementary material for: Effectiveness of the BNT162b2 (Pfizer-BioNTech) and the ChAdOx1 nCoV-19 (Oxford-AstraZeneca) vaccines for reducing susceptibility to infection with the Delta variant (B.1.617.2) of SARS-CoV-2
Source: BMC Infect Dis. 2022 Mar 20;22:270. doi: 10.1186/s12879-022-07239-z (PMC8934524; doi:10.1186/s12879-022-07239-z)
Supplement: Supplementary file 1 — Additional file 1. Population demographics, MCMC output (parameter estimates, trace plots and posterior distributions) for all models and log likelihood profile for infectiveness. [file 12879_2022_7239_MOESM1_ESM.pdf]

Additional File 1: Effectiveness of the BNT162b2 (Pfizer-BioNTech) and the ChAdOx1 nCoV-19 (Oxford-AstraZeneca) vaccines for reducing susceptibility to infection with the Delta variant (B.1.617.2) of SARS-CoV-2

Karan Pattni[1], Daniel Hungerford[2], Sarah Adams[3], Iain Buchan[4], Christopher P Cheyne[5], Marta García-Fiñana[5], Ian Hall[6,7], David M Hughes[5], Christopher E Overton[6], Xingna Zhang[4], Kieran J. Sharkey[1,*]

[1]Department of Mathematical Sciences, University of Liverpool, Liverpool, UK

[2]Centre for Global Vaccine Research, Institute of Infection, Veterinary and Ecological Sciences, University of Liverpool, Liverpool, UK

[3]Graphnet Health, Milton Keynes, UK

[4]Institute of Population Health, University of Liverpool, Liverpool, UK

[5]Department of Health Data Science, University of Liverpool, Liverpool, UK

[6]Department of Mathematics and School of Health Sciences, University of Manchester, Manchester, UK

[7]Joint Universities Pandemic and Epidemiological Research, Manchester, UK

[*]Corresponding Author: Kieran J. Sharkey, Department of Mathematical Sciences, University of Liverpool; +44 (0)151 794 4023 — kjs@liverpool.ac.uk

S1 Demographics for Population of Interest

Table S1.1: Demographics for population of interest.

Characteristic	Variable	Summary
Number of participants		2,730,111
Age (in years)	Recorded	2,730,111
	Unrecorded	0
	Mean (standard deviation)	42.1 (23.7)
	Median (Q1, Q3)	42.0 (23.0, 61.0)
	Range	0.0-109.0
Sex	Recorded	2,730,029
	Unrecorded	82
	Male	1,360,509 (49.8%)
	Female	1,369,520 (50.2%)
Ethnicity	Recorded	2,186,803
	Unrecorded	543,308
	Black, African, Black British, or Caribbean	19,674 (0.9%)
	Asian or Asian British	37,353 (1.7%)
	Another ethnic group	83,079 (3.8%)
	Mixed or multiple ethnic group	47,322 (2.2%)
	White	1,999,375 (91.4%)

Table S1.2: Vaccine doses administered by age group as extracted on 3rd Sep 2021, including those who died prior to having the vaccine or those who moved into or out of the area over the time period. No missing values due to the definition that no recorded values means no doses administered.

Age Group (years)	Total	No Doses	One Dose	Both Doses
$<$18	518,696	495,612 (95.5%)	20,040 (3.9%)	3,044 (0.6%)
18-39	772,847	274,896 (35.6%)	115,213 (14.9%)	382,738 (49.5%)
40-59	678,147	111,745 (16.4%)	30,041 (4.4%)	536,361 (79.1%)
60-79	545,019	43,884 (8.1%)	8,515 (1.6%)	492,620 (90.4%)
$\geq$80	158,964	22,810 (14.3%)	3,720 (2.3%)	132,434 (83.3%)
All	2,730,111	954,189 (35.0%)	178,699 (6.5%)	1,597,223 (58.5%)

S2 MCMC Parameter Estimates, Trace Plots and Posterior Distributions

Table S2.1: Parameter median estimates and 95% credible interval for all model fits. Notation is as follows β: Transmission rate, e_{ij}: Effectiveness of i doses of vaccine j, Initial I: Initial number of unvaccinated infected individuals, Initial I_{ij}: Initial number of infected individuals who have received i doses of vaccine j, μ_i: Infectiveness of an infected individual with i doses of either vaccine. For number of doses we have $i \in \{1, 2\}$ and for vaccines $j \in \{A, P\}$ for Oxford-AstraZeneca and Pfizer-BioNTech respectively.

Parameter	Core Model ($\gamma = 1/7$)	Sensitivity to γ ($\gamma = 1/3$)	Sensitivity to γ ($\gamma = 1/11$)	Double Recovered ($\gamma = 1/7$)
β	0.406<br>$[0.3893, 0.4425]$	0.7581<br>$[0.7239, 0.8146]$	0.3104<br>$[0.2953, 0.3448]$	0.4303<br>$[0.4121, 0.4702]$
e_{1A}	0.3851<br>$[0.3425, 0.426]$	0.3528<br>$[0.3117, 0.3918]$	0.408<br>$[0.3624, 0.4515]$	0.3637<br>$[0.3194, 0.4058]$
e_{2A}	0.6402<br>$[0.614, 0.6647]$	0.6618<br>$[0.6378, 0.6843]$	0.6247<br>$[0.5964, 0.6513]$	0.633<br>$[0.6066, 0.6582]$
e_{1P}	0.1954<br>$[0.1041, 0.2809]$	0.263<br>$[0.1815, 0.3399]$	0.1436<br>$[0.0452, 0.2353]$	0.1834<br>$[0.0914, 0.2692]$
e_{2P}	0.8392<br>$[0.8212, 0.8559]$	0.8425<br>$[0.8254, 0.8583]$	0.8369<br>$[0.8181, 0.8549]$	0.8365<br>$[0.8183, 0.8534]$
Initial I	54.0151<br>$[48.4328, 59.5435]$	46.6017<br>$[40.3953, 52.3173]$	56.2925<br>$[50.9526, 61.6423]$	54.0704<br>$[48.4778, 59.5674]$
Initial I_{1A}	14.8932<br>$[10.3012, 19.8382]$	12.8806<br>$[8.042, 18.6471]$	15.6489<br>$[11.369, 20.1298]$	14.8604<br>$[10.2687, 19.7842]$
Initial I_{2A}	2.4775<br>$[0.93, 4.7359]$	2.5871<br>$[0.8709, 5.4459]$	2.6046<br>$[1.0844, 4.7457]$	2.4337<br>$[0.9037, 4.739]$
Initial I_{1P}	0.7469<br>$[0.1234, 2.0681]$	1.0026<br>$[0.1587, 2.9233]$	0.6512<br>$[0.1119, 1.7435]$	0.7594<br>$[0.1288, 2.1112]$
Initial I_{2P}	2.9717<br>$[1.3856, 5.1267]$	3.5131<br>$[1.409, 6.6521]$	2.8499<br>$[1.4196, 4.7717]$	2.9786<br>$[1.3647, 5.1574]$
μ_1	0.8836<br>$[0.4464, 0.9955]$	0.8809<br>$[0.5336, 0.9949]$	0.864<br>$[0.3271, 0.9951]$	0.8809<br>$[0.4259, 0.9957]$
μ_2	0.0545<br>$[0.0021, 0.2734]$	0.0787<br>$[0.0031, 0.3343]$	0.0592<br>$[0.002, 0.309]$	0.0556<br>$[0.0022, 0.2809]$

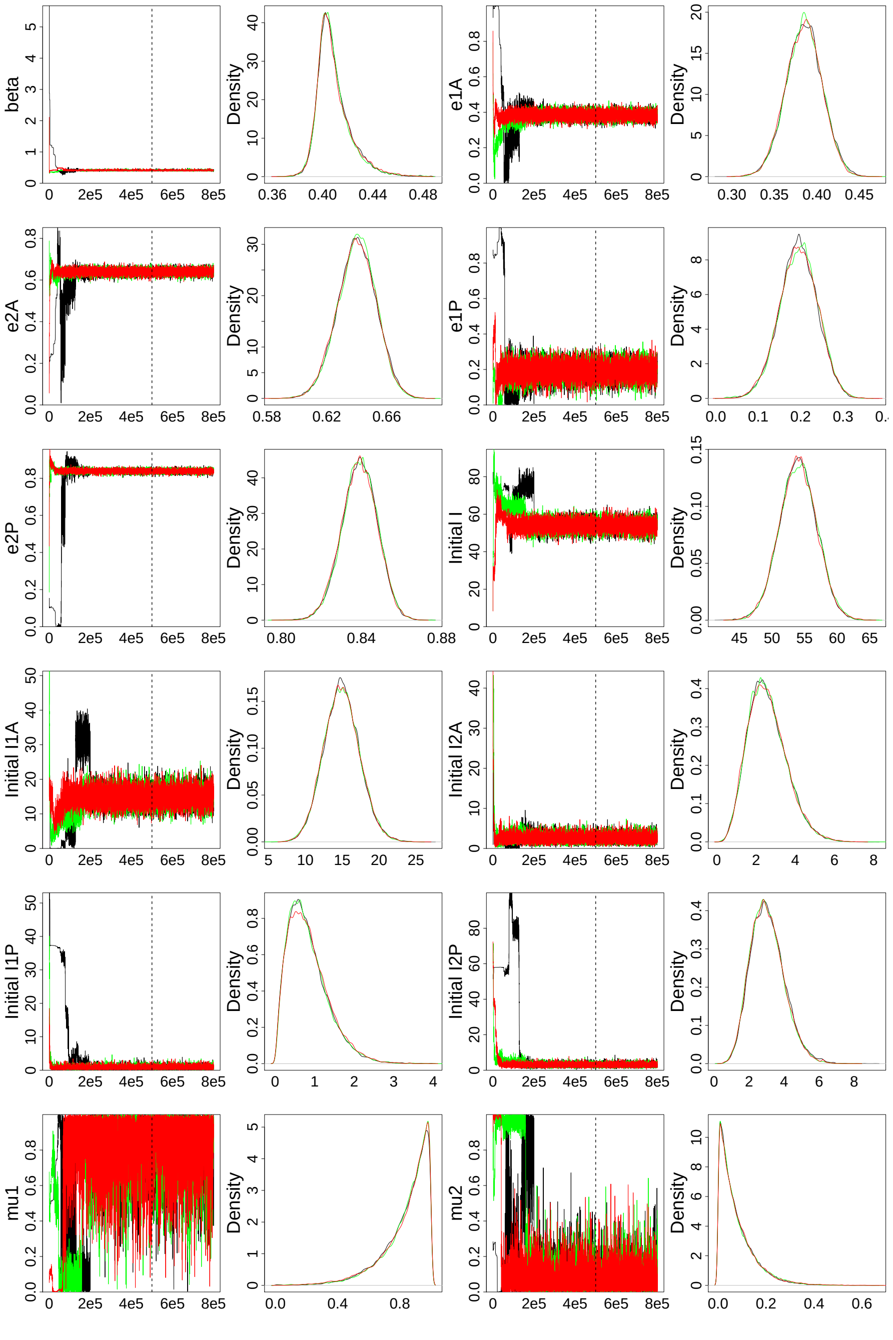

Figure S2.1: MCMC trace and posterior distributions for Core Model fit.

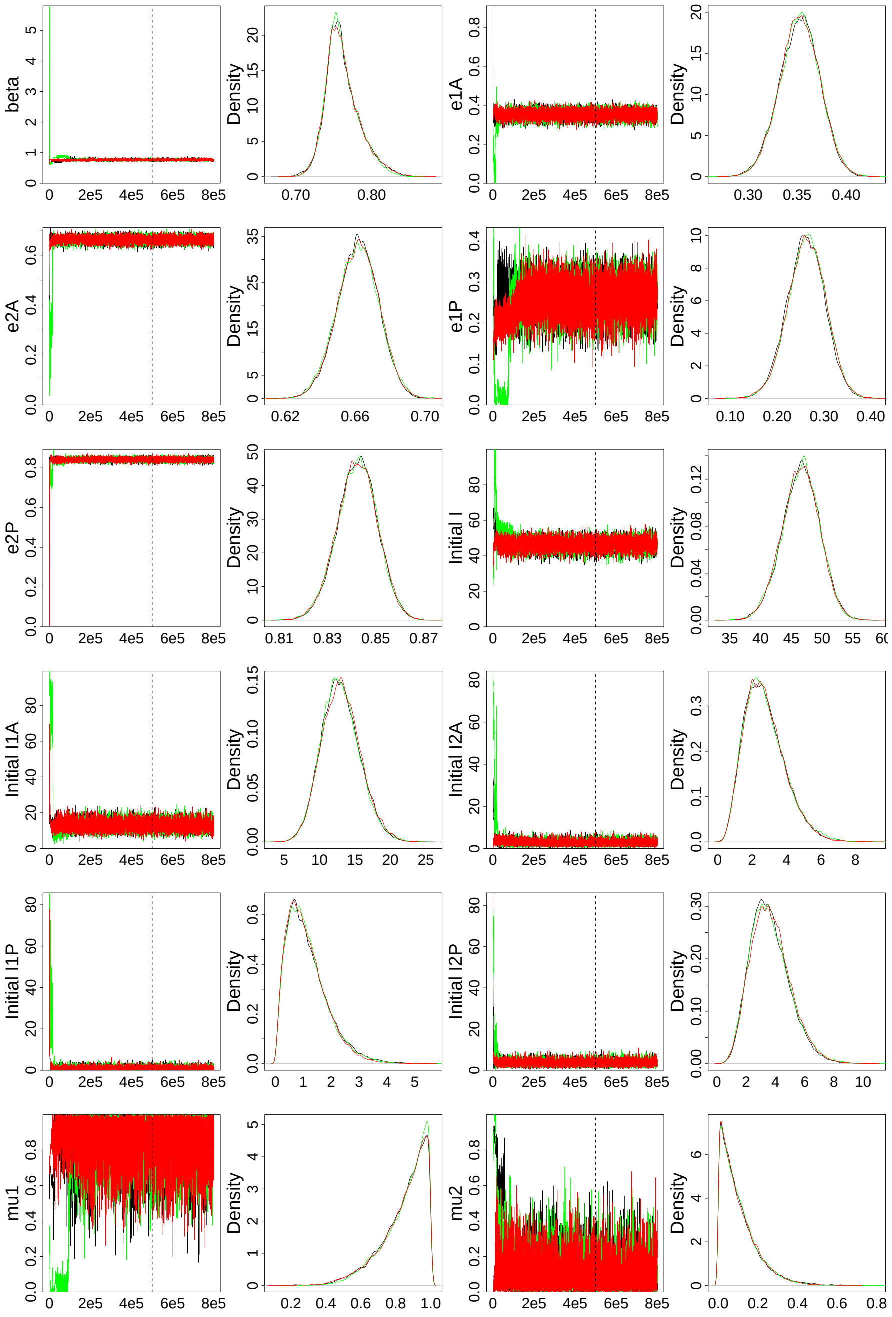

Figure S2.2: MCMC trace and posterior distributions for Sensitivity to γ ($\gamma = 1/3$) fit.

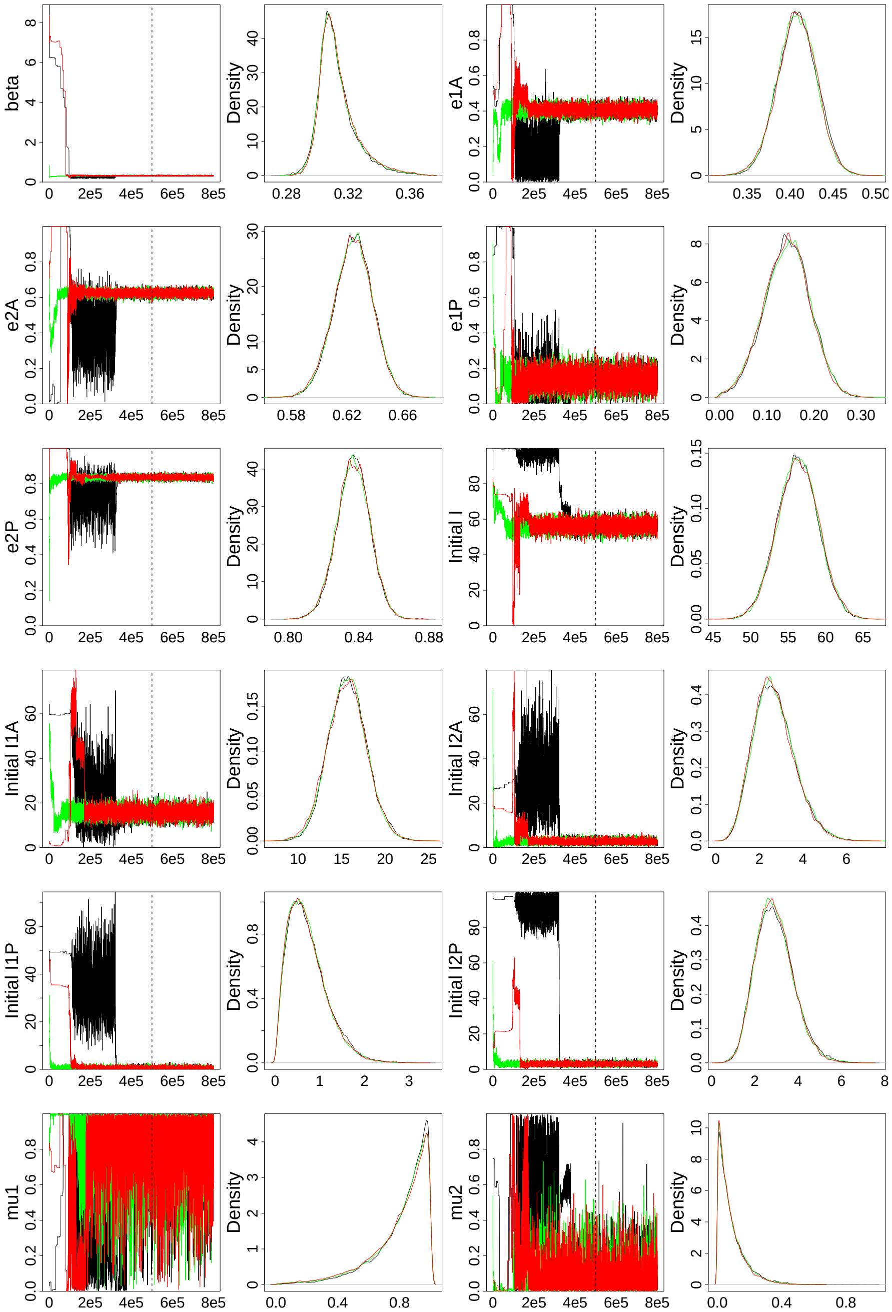

Figure S2.3: MCMC trace and posterior distributions for Sensitivity to γ ($\gamma = 1/11$) fit.

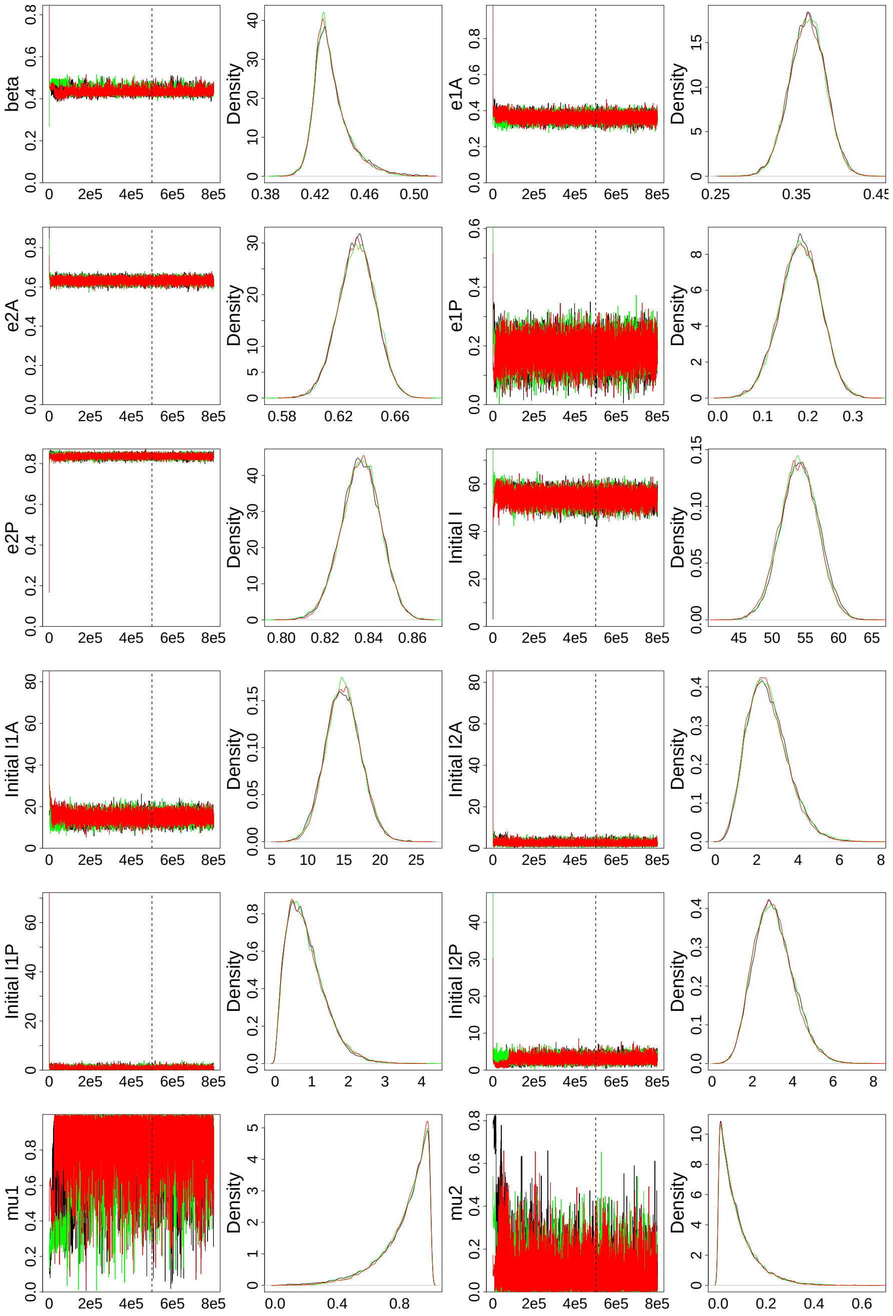

Figure S2.4: MCMC trace and posterior distributions for Double Removed fit.

S3 Log Likelihood Profile

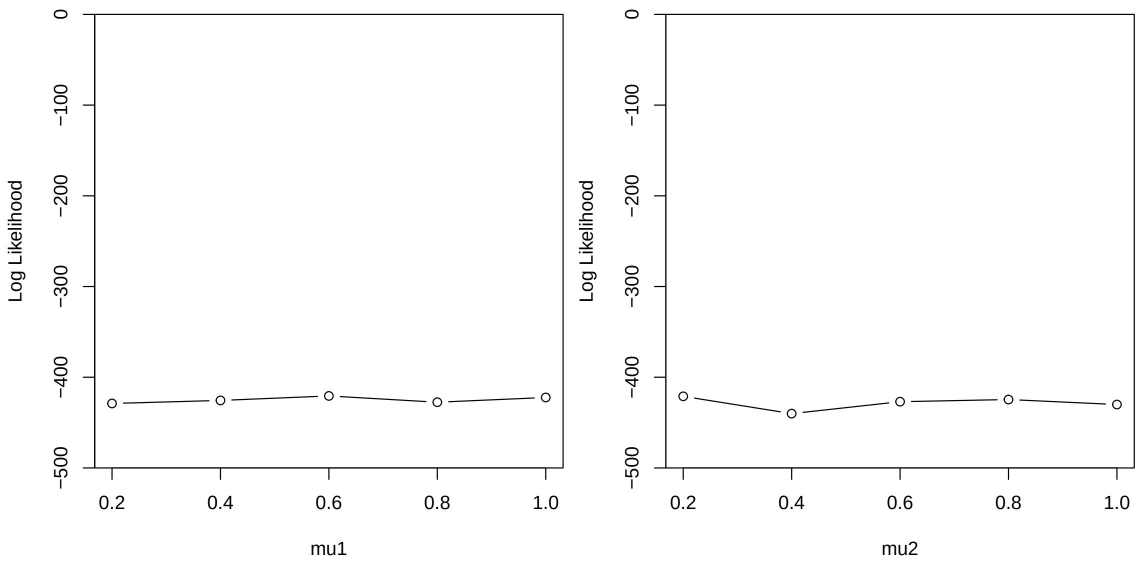

Figure S3.1: Log likelihood profiles for infectiveness (μ_i). To construct the log likelihood profile, we fix the value of μ_i and the find the optimum values of all other parameters (max likelihood) by fitting the model using MCMC as described in the main text. Doing this for all values of μ_i gives the likelihood profile. A flat profile implies alternating the value of μ_i does not improve the fit.
